# FPwatch: Facility‐based Survey Data for Family Planning Market Analysis in Five FP2020‐focus Countries

**DOI:** 10.1111/sifp.12077

**Published:** 2018-11-19

**Authors:** Saleh Babazadeh, Katherine Thanel, Danielle Garfinkel, Christina Riley, Jane Bertrand, Bryan Shaw

## Abstract

This article describes datasets for the FPwatch Project, a comprehensive facility‐based family planning survey conducted by Population Services International in five countries in Africa and Asia from 2015 to 2017. Contents cover research design and background, methodology, sample selection, data collection, an overview of FPwatch indicators, and quality assurance measures taken. These datasets from the Democratic Republic of Congo, Ethiopia, India, Myanmar, and Nigeria complement other facility‐based family planning surveys and are unique in their large‐scale, standardized methodology, and comprehensive sampling approach. In addition, all datasets but Myanmar (private only) include both private and public facilities, a feature that gives a more complete picture of the family planning supply environment. Because of these factors, the data is well suited to inform global family planning efforts.

Expanding access to modern contraception is a primary aim of global family planning efforts; however, contraceptive supply environments are little known in many low‐income settings. There is a lack of rigorous data on contraceptive availability at the outlet‐level with which to inform policies. A better understanding of the availability of modern contraception and market forces affecting men's and women's access to modern contraception in these countries is critical to reach the ambitious global FP2020 goal of expanding access to family planning information, supplies, and services to an additional 120 million women and girls in the world's poorest countries by 2020 and to reach national FP2020 goals (Askew and Brady 2013; Brown et al. 2014; Harrison and Do 2016). To address this need, Population Services International (PSI) implemented FPwatch, a multicountry research project conducted from 2015 to 2017 designed to provide timely, relevant, and high‐quality evidence on modern contraceptive commodities and services. The FPwatch study was designed based on a total market approach, a consumer‐focused systems lens in which the interplay of all sectors—public, socially marketed, and private (nonprofit and for‐profit commercial)—is considered to ensure health care delivery to all segments of the population. The goal is to make sure that those in need are reached with the appropriate products and services: that those in the poorest communities receive free products and services, those with slightly greater resources benefit from partially subsidized products and services, and those with a greater ability to pay are able to purchase products and services from the commercial sector (Meekers, Haynes, and Kampa 2016). To better understand how to meet these needs and to deliver high‐quality data, FPwatch examined the supply side of the total contraceptive market using facility‐based surveys in five FP2020‐focus countries: the Democratic Republic of the Congo (DRC), Ethiopia, India, Myanmar, and Nigeria. FPwatch was funded by the Three Millennium Development Goal (3MDG) Fund in Myanmar and by the Bill & Melinda Gates Foundation in the remaining four countries.

Similar to other efforts to monitor progress toward FP2020 goals, the FPwatch methodology produced a full picture of the market for contraceptive commodities and services within a geographic area. While most facility‐based surveys are designed to collect information on modern contraceptive commodity and service availability among a selection of facilities, FPwatch collected additional data on price, stockouts, and the volume of products and services distributed, as well as other family planning (FP) service quality information (Tumlinson 2016). A major distinction of FPwatch, compared to other facility‐based surveys, is that FPwatch conducted a full census of all facilities potentially offering FP products or services within selected geographic areas, including those outside the formal health system such as itinerant and/or mobile drug vendors, unregistered drug outlets, and traditional medicine providers. The extent of all outlet types visited during the FPwatch surveys are detailed elsewhere (FPwatch group 2016a, 2016b, 2016c, 2017a, 2017b; Riley et al. 2018). The comprehensive census approach within sampled geographic areas allowed for unique estimates of market composition (e.g., what is the proportional availability of contraceptive methods across a range of facility types?) and market volume (e.g., how is the volume of contraceptive methods distributed across and within all types of facilities?).

The volume of each contraceptive method can be converted to its respective share of Couple Years of Protection (CYPs)^1^ distributed by each facility, which allows for analysis of market share. This information is vital for global and country‐level decision‐making, funding management, and policy design. The tracking of market composition and volume complements the data available in existing facility surveys such as the Performance, Monitoring, and Accountability 2020 (PMA2020) and Service Delivery Point (SDP) surveys (PMA2020 2017; Zimmerman et al. 2017). FPwatch adds value in the magnitude of its sample size, unique indicators, systematic census approach in selected areas, and comprehensive, outlet‐specific, public and private sector data, which illustrate a full picture of the FP supply environment and generate the necessary information to inform evidence‐based decision‐making for the FP market. Table 1 illustrates a comparison of indicators across similar, large‐scale facility‐based surveys.

**Table 1:** Comparison of questions and computable indicators of quality from facility‐based surveys (adapted from Tumlinson 2016)

Indicator	SA	QIQ	SARA	DHS‐SPA	MLE	PMA 2020	FPwatch
Availability of commodities							
Does this facility provide the following methods?	Yes	Yes	Yes	Yes	Yes	Yes	Yes
Are they currently available/observed?	Yes	Yes	Yes	Yes	Yes	Yes	Yes
Have there been any stock‐outs in last 3/6/12 months?	Yes	Yes	Yes	No	Yes	Yes	Yes
Outlet type	Yes	Yes	Yes	Yes	Yes	Yes	Yes
Is family planning integrated in other health services?	No	No	No	Yes	Yes	Yes	No
Infrastructure							
Availability of sterilizing equipment, gauze, gloves, blood pressure cuff, specula, bed, etc.	Yes	Yes	Yes	Yes	Yes	Yes	Yes
Availability of private exam rooms?	Yes	No	Yes	Yes	Yes	Yes	Yes
Availability of family planning guidelines?	No	Yes	Yes	Yes	Yes	No	Yes
Quality assurance system in place	Yes	Yes	Yes	Yes	Yes	Yes	Yes
Services readiness							
Are there staff providing FP services?	Yes	No	Yes	Yes	Yes	Yes	Yes
Are the staff trained for FP?	Yes	No	Yes	Yes	Yes	No	Yes
Required equipment for FP methods available?	Yes	Yes	Yes	Yes	Yes	Yes	Yes
Market analysis							
Method market composition	Yes	Yes	Yes	Yes	Yes	Yes	Yes
Markup price of modern FP methods	No	No	No	No	No	Yes	Yes
Volume of FP methods distributed/sold	No	No	No	No	No	No	Yes
Modern FP method market share by brand	No	No	No	No	No	No	Yes

SA = Situation Analysis, developed by the Population Council.

QIQ = Quick Investigation of Quality, MEASURE Evaluation.

SARA = Service Availability and Readiness Assessment, World Health Organization DHS‐SPA: Demographic and Health Survey‐Service Provision Assessment.

MLE = Measurement, Learning & Evaluation Project, UNC‐Chapel Hill.

PMA 2020 = Performance Monitoring and Accountability 2020.

Standardized tools and approaches were applied to produce comparable data across the five countries. FPwatch surveys generated nationally‐ or subnationally‐representative data from a cross‐section of public and private outlets—any point of delivery of a modern contraceptive commodity or service—and applied the same rigorous methodology and data quality standards as its malaria‐focused sister project, ACTwatch (Shewchuk et al. 2011; O'Connell et al. 2013). FPwatch did not include public outlets in Myanmar because of a concurrent public facility‐based survey funded by UNFPA. FPwatch countries were selected based on their large populations and high unmet need—priorities for reaching FP2020 goals. The modern contraceptive supply environments in these countries are in varying stages of development, thus the survey results provide a set of valuable insights for regulatory organizations and policymakers on the availability of modern contraceptive commodities and services to all segments of the population. A Technical Advisory Group of family planning experts was convened to provide guidance on indicator development for the FPwatch project. FPwatch worked in coordination with PMA2020 and FP2020 to prioritize filling data gaps and to harmonize indicators for comparability.

## FPWATCH DATA AND INDICATORS

The FPwatch study collected information at both the outlet and product levels. At the outlet level, every potential delivery point for modern contraception was approached (ranging from mobile vendors/kiosks to tertiary hospitals) and information was collected on the type of outlet and service characteristics. At each outlet with modern contraception available, a comprehensive audit of all unique contraceptive products was conducted. For the audit, information was recorded on all forms of short‐acting contraception, long‐acting reversible contraception (LARCs), and permanent methods down to the level of unique brands to provide data on availability, manufacturing information, price, stock‐outs, and volume distributed in the previous one month. In addition, the study collected information on all associated services for contraceptive injections, LARC insertions, and male/female sterilizations, including service quality questions on availability of qualified providers and a minimum package of essential equipment needed to conduct the service (MEASURE Evaluation 2012). All variables collected for the survey are available in Table 2 and a full list of FPwatch indicators is available in FPwatch reports at http://www.psi.org/research/library.

**Table 2:** List of key variables in the dataset

**Data elements**	**For services**:
Outlet information Outlet type Geography Outlet unique identifier Urban or rural State/region code Township code	Full price of product (service fee + commodity price) Reported availability of FP services (injections, implant insertion, IUD insertion, male vasectomy, and/or female tubal ligation) Provision of services on the day of the survey Staff training on FP counseling last 2 years Staff training on service provision (for each service type reported available) in last 2 years FP provider health qualification Name of training organizations Total number of procedures provided in the past month, by method type Perform services for commodities bought elsewhere Type of staff that perform each procedure Private area available to perform the service (contraceptive injections, implant procedures, IUD insertions, and/or male/female vasectomy) For injections—availability of disposable needles For implant insertion—availability of trocar, iodine, scalpel, forceps For IUD insertion—availability of examination table, iodine, tenaculum, speculum, uterine sound, string retriever Type of female tubal ligation performed (mini‐laparotomy, laparoscopy, trans‐cervical) For female tubal ligation and male vasectomy—availability of surgical table, blood pressure app, lidocaine, sterile needle w/syringe, scalpel, uterine elevator, tubal hook, ringed clamp, dissecting forceps
**For modern contraceptive products**	
Most common brand Per/unit wholesale purchase price Per/unit retail selling price In stock on the day of the survey In stock in last 3 months Number of products audited, by type Distributed through dispensary/pharmacy/outpatient Product number Brand name Generic name Manufacturer Country of manufacture Package size (blister packages) For male and female condoms: Number distributed in the last 7 days Amount sold/distributed in the last month (blister packages, individual tablets, or product units) Source of information for amount sold Stocked out of brand at any point in the past 3 months Supplier of brand	

### Sample Selection and Size

Sampling procedures were discussed with both national Ministries of Health and the FPwatch Technical Advisory Group to reflect local priorities for information. The FPwatch data collection teams surveyed a representative sample at the national and regional levels in Myanmar and Nigeria, and at the regional level in the DRC (Kinshasa and Katanga provinces), Ethiopia (Addis Ababa; Amhara; Oromia; Southern Nations, Nationalities, and Peoples’ regions), and India (Bihar and Uttar Pradesh states). In the DRC, geographic units were organized into rural and urban strata, and in India and Myanmar an additional stratum for metro areas was included. See Table 3 for sampling information by country.

**Table 3:** Characteristics of data collection by country for FPwatch project

Country	Dates of datacollection	Level at which dataare representative	Outlets screened	Outlets interviewed	Contraceptives audited	Response rate^a^
Ethiopia	July–August 2015	Four regions: Addis Ababa, Amhara, Oromia, SNNP^b^	8,299	2,082	6,998	>99%
Nigeria	August–October 2015	National: All six geopolitical zones^c^	14,269	2,524	4,332	>98%
DRC^d^	October–December 2015	Kinshasa (urban/rural) and Katanga (urban/rural)	2,445	1,297	2,238	>99%
Myanmar	February–May, 2016	National; metro, urban, and rural	19,772	7,791	7,743	>99%
India	June–September, 2016	Uttar Pradesh and Bihar states	36,723	4,083	14,902	>98%
**Total**			**81,508**	**17,777**	**36,213**	**>99%**

a Out of interviewed outlets.

b Southern Nations, Nationalities, and Peoples’ region.

c In Nigeria, certain localities were excluded in the two insecure North East states of Borno and Yobe.

d In 2015, the DRC's 11 original provinces were divided into 26; FPwatch used the pre‐2015 boundaries in province selection.

**Figure 1:**
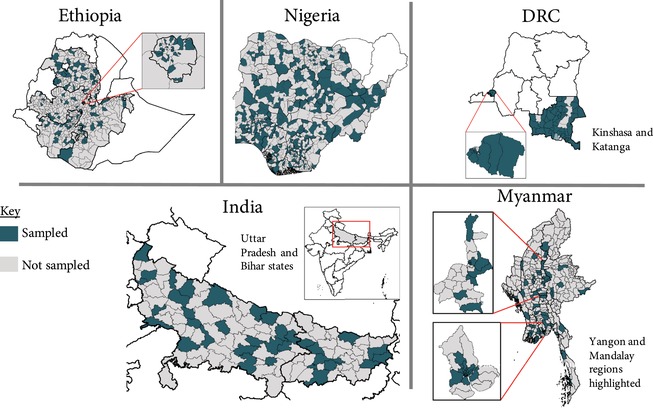
Maps of sampling areas by FPwatch country NOTE: Maps created by Population Services International.

Two‐stage cluster sampling was conducted in each region/country, with the exception of metro areas where single‐stage sampling was used to fit administrative boundaries in areas with high population density. At the first stage, within each region, administrative areas corresponding to districts were sampled using probability‐proportional‐to‐size (PPS) sampling. At the second stage, within selected districts, a list of geographic units with approximately 10,000 to 15,000 inhabitants was used as a sampling frame. Population size acted as a proxy for outlet density because accurate estimates on the total number of outlets in each administrative unit were not typically available. Sample size requirements were based on back‐calculations from PMA2020 survey estimates of outlets with three or more modern methods of contraception available (PMA2020 2017). The number of outlets surveyed was monitored throughout data collection to ensure that sample size estimations were adequate. Additional primary sampling units were added to the sampling frame using PPS if the number of outlets surveyed was lower than expected. Non‐response rates were generally low, ranging from 1 percent in the DRC, Ethiopia, and Myanmar to 2 percent in India and Nigeria. The sampling strategy for each country is described in additional detail in respective country reports (FPwatch group 2016a, 2016b, 2016c, 2017a, 2017b), which can be found at http://www.psi.org/research/library. Figure 1 shows the maps of sampled areas in each country.

### How and When Data Were Collected

From 2015–16, FPwatch completed five outlet surveys in FP2020‐focus countries. Data collection typically lasted six to eight weeks, and dates of data collection are provided in Table 3 for each country/region. Data collectors met with local authorities to determine administrative boundaries and obtain facility lists if available. The data collection teams walked or drove the boundaries of a selected area, used existing maps or created sketch maps, and then conducted a full census of outlets within the boundaries, approaching all known outlets and identifying nontraditional, unregistered, mobile, or recently opened outlets.

Every public‐ and private‐sector outlet, with the exception of those that catered to specialized populations such as military hospitals or those that primarily distributed contraception for prevention of sexually transmitted infections as opposed to pregnancy prevention (e.g., bars, hotels, brothels), were approached for inclusion in the survey. All outlets were screened with a short questionnaire and all eligible outlets were interviewed with the full‐length survey. Outlets must have met one of the following criteria to be eligible for a full interview and product audit: (1) currently stocking at least one brand of a modern contraceptive method including: oral contraceptives, emergency contraceptives, injectable contraceptives, contraceptive implants, or intrauterine devices (IUDs); (2) stocked one or more of these methods in the previous three months (even if currently stocked out); or (3) currently offering provider‐dependent contraceptive services including contraceptive injection, implant insertion, IUD insertion, or male/female sterilization services. For outlets stocking other forms of modern contraception such as male condoms, but not meeting the above criteria, a mini‐audit was conducted collecting information on available brands, prices, and volume distributed in the previous month.

Public sector outlets included hospitals, health centers, and community health workers. Health centers included various levels of public facilities, from the rural health post or sub‐center to higher‐level facilities. Private sector outlets typically included private for‐profit hospitals, clinics, pharmacies, drug shops, and general retailers, but also moonlighting health workers and itinerant drug vendors (in Myanmar) and traditional medicine clinics (in India).

Each outlet's senior‐most available staff was approached to complete the survey. At all eligible outlets, providers were invited to join the study after giving verbal informed consent. Data collectors asked to see all modern contraceptive products in stock (male condoms, female condoms, oral contraceptives, emergency contraception, injectables, implants, IUDs, CycleBeads, vaginal patches, rings, foaming tablets, and diaphragms) and used paper questionnaires or electronic tablets to complete audits of relevant product information including: brand name, generic name, active ingredients and corresponding strengths, and manufacturer name and country. For each unique brand, interviewers asked providers about volume distributed during the previous month, stock‐outs during the previous three months, source of supply, and retail and wholesale price. For information on volume, interviewers recorded whether responses were self‐report or taken from written records. At outlets where provider‐dependent services (e.g., IUD insertions) were reportedly offered, questions were asked about provider certification and necessary equipment in addition to commodity questions. The coordinates of each outlet were recorded using Global Positioning System (GPS) devices.

### Data Quality

In each country, data collection was conducted by a local research agency or a local PSI affiliate. Data collectors undertook a two‐week training covering all aspects of data collection. Data collectors were a mix of male and female university graduates, fluent in the predominant languages of the regions in which they were conducting data collection, and with experience conducting large‐scale surveys. Only data collectors meeting a high standard of competency during training were selected to participate in data collection. Exceptional scorers were selected for team supervisor and quality‐control positions. Additional days of training for these positions were provided covering personnel management, census procedures, questionnaire review, and outlet follow‐up procedures. Quality controllers reviewed all completed surveys each evening in order to ensure data collection accuracy and adherence to skip patterns. In addition, quality controllers re‐interviewed a random sample of 10 percent of approached outlets for quality assurance. Data were double entered into a Microsoft Access database by trained data‐entry personnel. Several staff from PSI‐Washington, DC and the local PSI affiliate accompanied teams throughout data collection on a rotating basis to provide an additional level of quality control.

### Data Formats, Location, and Access

All study datasets are available at https://dataverse.harvard.edu/, and can be obtained after submitting affiliation and brief description of intended use to PSI. Raw data are available in English, in csv, xls, or dta format. Data were cleaned during and after data collection, for example by deleting duplicate entries or removing outlets that did not fall within geographic limits. However, missing and extreme values were not modified at this stage and incomplete surveys were retained. Data requestors have access to Stata 13 dataset files on the Harvard Dataverse site. Detailed data analysis instructions, codebooks, and reference documents are also available upon request. To protect outlet identity, GPS coordinates and other identifying variables have been removed (Anyanti et al. 2017; Shaw, Girma, Tamene et al. 2017; Shaw, Onema, Mpanga et al. 2017; Shaw, Sharma, Ponnusamy et al. 2017; Shaw, Me Thet, Aung, et al. 2017).

Country reports were prepared based on FPwatch indicators and are available at www.fpwatch.info. These comprehensive reports include full analysis tables with estimates of primary indicators. In addition, summary briefs for each country and region are available with high‐level findings and additional country context.

### Data Use

FPwatch offers a comprehensive set of materials to support use of FPwatch data. Specific guidelines for researchers interested in analyzing FPwatch data are outlined in the document “FPwatch Project Guidance Document,” which is available for download at: https://dataverse.harvard.edu/. Complementary codebooks for each dataset with a brief explanation for each variable and survey questionnaires are also available at this site. The guidance document describes indicators in detail and reviews analysis considerations for couple years of protection, sampling weights, and dataset structure. It also includes additional detail on survey design, outlet definitions, history of data cleaning and handling of missing values, dataset structure, booster samples and weighting specifications, and sample size calculations. Suggested citations are available within the datasets.

### Value of the Data

The strength of FPwatch lies in its unique indicators and large‐scale, standardized, census‐approach within selected areas.

- With 80,000+ outlets screened across five countries, FPwatch includes comprehensive, precise data on contraceptive availability, volume distributed, market composition, and price at the outlet level. FPwatch surveyed a large diversity of outlet types, including previously unmeasured outlets for which estimates were not available, such as itinerant drug vendors and informal outlets, providing a clear understanding of the roles of these outlets. Only a survey with a large sample size, such as FPwatch is able to obtain precise estimates for each of these outlet types. The data are nationally and regionally representative in Myanmar and Nigeria, and regionally representative in the DRC, Ethiopia, and India.
- As the only facility‐based survey that provides a full census‐approach within selected geographic areas across all sectors for both contraceptive commodities and services, FPwatch is able to provide a full picture of the market; this denominator gives confidence in answering a wide range of research questions related to market composition and market share, indicators that have not been possible to measure in smaller‐scale surveys. The ability to calculate with precision the Couple Years of Protection (CYPs) contribution of each outlet type makes FPwatch unparalleled in its data contribution in the context of tracking FP2020 goals, and provides a unique complement to other facility‐based contraceptive health surveys that only examine selected types of outlets.
- Large‐scale facility‐based surveys often have a limited focus, with an emphasis solely on provider services or the public sector, and may not be feasible to conduct routinely (Hozumi et al. 2006). As the largest, most robust census‐based family planning facility survey for commodities and services to date, FPwatch incorporates many of the aspects examined in existing surveys, while expanding the scope of examination to include a full product audit and provider interviews for a more wide‐reaching assessment. The breadth of FPwatch's sample and range of outlets identified are hallmarks of the comprehensive nature of the study.
- The countries were selected based on their FP2020 status and their potential to benefit from data tracking around national FP2020 commitments.
- In terms of sector‐specific data, FPwatch demonstrates added value in its ability to illustrate the private sector market and contribute comprehensive, outlet‐specific private sector data. In developing countries, where information on the private sector is largely missing, or not required or regulated (Ahmadi et al. 2013; Phalkey et al. 2017), FPwatch data allow for comparability within and across countries, and paint a clear picture of the contraceptive market for program and policy implementers. In filling the gap in private sector knowledge, FPwatch data highlight missed opportunities in a country's overall modern contraceptive market such as the potential access to modern contraception offered via the ubiquity of drug shops in Nigeria and DRC (Riley et al. 2018).
- FPwatch's standardized approach allows for comparability of total markets. The success of countries with strong markets can provide lessons learned and act as a roadmap for identifying opportunities such as high‐impact outlet types in countries with less developed markets (Thanel et al. 2018).

## Footnotes

1 CYP is the estimated protection provided by contraceptive methods during a one‐year period, based upon the volume of all contraceptives sold or distributed free of charge to clients during that period. From: https://www.usaid.gov/what-we-do/global-health/family-planning/couple-years-protection-cyp.
